# Effects of Organic Substrate Amendments on Selected Organic Fractions and Biochemical Parameters under Different Soils

**DOI:** 10.1155/2024/9997751

**Published:** 2024-09-09

**Authors:** Nguyen Do Chau Giang, Tran Van Dung, Nguyen Minh Dong, Nguyen Minh Phuong, Vu Van Long

**Affiliations:** ^1^ Faculty of Soil Science College of Agriculture Can Tho University, Can Tho 94100, Vietnam; ^2^ Faculty of Natural Resources - Environment Kien Giang University, Kien Giang 91752, Vietnam

## Abstract

The application of organic substrates can affect soil respiration, dehydrogenase (DH-ase) activity, dissolved organic carbon (DOC), and humic acid (HA) fractions. This study aimed to evaluate the effects of five organic substrates in the organic fractions of degraded alluvial soil, acid sulfate soil, and sandy soils and the physicochemical properties of the soil. Soil samples were amended at a rate of 5 tons ha^−1^ with (1) water hyacinth compost, (2) sugarcane filter cake compost, (3) biogas sludge-rice straw compost, (4) vermicompost, and (5) sludge. The results showed that soil respiration and DH-ase activity increased rapidly within the first 5 days of incubation, while the concentrations of DOC and HA decreased throughout the incubation period. The highest respiration and DH-ase activity occurred after the application of vermicompost. DOC was found to be the highest in soils amended with sugarcane. The highest concentration of HA was observed with the application of sugarcane residues, regardless of the type of soil. The application of water hyacinth and biogas sludge stimulated cumulative HA only in the acid sulfate soil, while vermicompost improved HA only in the degraded soil. The largest stimulation in respiration and DH-ase activity was observed in degraded and sandy soils, regardless of the type of amendment. In the acid sulfate soil (3.7 mg·C·g^−1^), larger amounts of DOC and HA were observed than in both degraded (1.7 mg·C·g^−1^) and sandy soils (1 mg·C·g^−1^). However, DH-ase activity was the lowest in acid sulfate soil.

## 1. Introduction

The recycling of biological waste to organic fertilizers is a way to reuse nutrients and reduce environmental pollution. Various biological wastes such as biological municipal waste, animal excreta, and plant residues can be composted [1]. The application of composts or other organic substrates, such as sewage sludge is a current environmental and agricultural practice for maintaining soil organic matter, reclaiming degraded soils, and supplying plant nutrients [2, 3]. An adequate supply of nitrogen (N), phosphorus (P), potassium (K), and other essential nutrients is necessary to maintain crop productivity.

For many decades, farmers have used mature composts to maintain soil fertility and improve plant yields. After being engulfed in soils, organic substrates can be mineralized and then nutrients can be released in a more readily available form to plants [4]. Immature or uncomposted organic substrates still have high microbial activity and might contain toxic compounds that can immobilize mineral nutrient elements in soils and harm plants. There are several ways to treat biological waste. They can be aerobically composted or anaerobic, such as by fermentation in a biogas reactor. The composition of biological wastes significantly influences their organic matter and nutrient content as a result of mineralization processes during the treatment process or after amendment in the soils. Immature compost or biogas sludge contains highly soluble organic carbon substances such as amino acids and carbohydrates [5, 6]. They can easily decompose after application to soils and make soils more anoxic. However, anoxic conditions can mobilize heavy metals in the soil. Immature compost or biogas sludge might also contain large amounts of intermediate organic products of decomposition, such as volatile fatty acids, alcohols, and phenols, which are toxic to plants. Therefore, in the usual agricultural practice, these organic substrates are applied some weeks before sowing to allow soil microorganisms to degrade labile organic matter and phytotoxic compounds and to release plant nutrients [7, 8].

A reduction in carbohydrates, hemicellulose, and cellulose during the composting process is usually accompanied by increased humification. Therefore, humification is an index of compost maturity. Humic substances are valuable components of compost. In additions, the humification process increases the content of alkyl C, aromatic C, carboxyl (-COOH), phenolic (-OH), and carbonyl (-CO) groups, which are beneficial to the physical and chemical properties of the soil and plant productivity. When the C : N ratio of compost is lower than 20, the mineralization of N in compost is dominated by microbial N immobilization processes, which facilitates the release of mineral N for plant use [1, 9]. The stability and maturity of organic substrates prior to soil application have shown to have strong effects on the biological and chemical properties of soils. Mature organic substrates can be defined as the stabilized and sanitized products of composting, which undergo a hot rotting process to decompose labile organic matter and are humified [10], and are consequently beneficial to plant growth.

However, depending on the composting facility and compost demand, organic substrates applied to soil can have different degrees of maturity, i.e., they can be taken after the biooxidative phase before maturation and can be slightly transformed during the biooxidative phase or even during the thermophilic phase. Biological decomposition depends on the degradation rate of a wide range of C compounds present in organic substrates (carbohydrates, amino acids, fatty acids, and lignin), as well as on their nutrient content [11]. Therefore, the effect of each organic substrate on soil properties depends on its composition. The influence of organic matter on soil properties depends on the amount, type, and size of added organic materials [12, 13]. The decomposition of organic matter in soils is dependent on microbial activity. The release of nutrients from applied organic substrate decreases exponentially with time as a result of decreasing carbon availability [14].

Microbial respiration, i.e., the amount of CO_2_ released from compost, is often used as an indicator of microbial activity and the maturity (stability) of compost [15, 16]. The high microbial activity of applied compost might also promote the degradation of soil organic matter (SOM). This is usually undesirable, as SOM is a very important component that determines the quality and fertility of soils [17–19]. The extent of biodegradation may also be reflected in the amount of dissolved organic C available during the decomposable period. The availability of carbon is essential for most microorganisms because carbon serves as an energy source for the synthesis of ATP. The main components of the organic matter are carbohydrates, proteins, lipids, and lignin. They differ in the ease with which microorganisms can decompose them by aerobic (oxidation) or anaerobic (reduction) mineralization processes.

Rapidly and slowly mineralizable portions of dissolved organic carbon (DOC) are a measure of labile and stable DOC and the respective mineralization rate constants [20, 21]. These labile organic compounds mineralize quickly in soil and are oxidized during degradation. Components that are more resistant to mineralization, such as aromatic and hydrophobic structures, can be assessed by UV absorbance to quantify the amount of humic acid in the soil. The biodegradability of DOC can be reduced due to its recalcitrance or inhibition of enzyme activity [22]. The capacity of microorganisms to assimilate organic matter depends on their ability to produce the enzymes needed for degradation of the substrate [23, 24]. The more complex the substrate is, the more extensive and comprehensive the enzyme system requires source, which is incorporated in the cells. In particular, the presence of ester bonds, aromatic rings, quarterly C, and tertiary N in macromolecular carbon substrates increases their stability and recalcitrance to microbial decay processes and plays a key role in the formation of more stable soil organic matter. On the other hand, water-soluble (hydrophilic) carbon substrates of low molecular weight are easily and rapidly mineralized. They are part of the labile C pool and are important for the release of plant nutrients. Since many enzymes respond immediately to changes in soil fertility status, they can be used as potential indicators of soil quality for sustainable management. For example, dehydrogenase plays an important role in the initial oxidation of soil organic matter and occurs only in viable cells; therefore, dehydrogenase activity is a sensible indicator for measuring the metabolic activity of microorganisms in soils [25].

In this study, we hypothesized that the application of organic substrates to different soils would differentially affect soil respiration (Res), dehydrogenase (DH-ase) activity, dissolved organic carbon (DOC), and humic acid (HA) fractions. To test this hypothesis, we evaluated five organic substrates in three different soils under laboratory incubation conditions.

## 2. Materials and Methods

### 2.1. Soil and Organic Substrates in This Study

Three different soil types used for this experiment included old alluvial soil (degraded soil), acid sulfate, and sandy soil. The soil was taken to a depth of 0–20 cm, air dried, homogenized, and sieved through a mesh of 2 mm before use.

Five organic substrates were used for the experiment: water hyacinth compost, sugarcane filter cake compost, biogas sludge-rice straw compost, vermicompost, and sludge (undigested slurry). The characteristics of the experimental soils and organic substrates are shown in Table 1.

### 2.2. Experimental Setup

#### 2.2.1. Respiration Experiment

The respiration experiment was designed with 22 treatments and four replications to determine microbial activity based on oxygen consumption (Table 2). To set up the experiment, a hole was made into the caps of the glass bottles using a drilling machine. Five grams of pure soil (control), pure compost, or 5 g of soil amended with a compost equivalent to 50 Mg·ha^−1^ was added to each bottle. After the addition of water, the soil or substrate mixtures were homogenized by using a stainless steel spoon. A 2-ml tube was inserted vertically into each bottle, into which approximately 2 pellets of NaOH were placed. The bottles were covered with rubber septa, sealed, airtight, and placed in a climate cabinet at a constant temperature of 27°C. These bottles contained the samples. The samples were incubated for 90 days. The change in internal pressure was recorded every day for each bottle.

Respiration activity was calculated from the pressure change using the following formula:

(1) $$\begin{array}{c} \text{pV}=\text{nRT}, \end{array}$$

where *p* is the pressure (hPa), V is the volume (L), *n* is the substance in mol, *R* is the universal gas constant, and *T* is the temperature (°K).

The following equation was used to calculate the daily oxygen consumption:

(2) $$\begin{array}{c} \text{AO}=\frac{\left(M_{R}\left(O_{2}\right)\right)}{\left(R\times T\right)}\times \frac{V_{\text{fr}}}{m_{\text{ts}}}\times ∆p, \end{array}$$

where *M*_*R*_(O_2_) is the molar mass of O_2_ (mg mol^−1^), *R* is the universal gas constant; *T* is the temperature of incubation in (°K), Δp is the decrease in pressure in the bottle (hPa), *V*_fr_ is the volume of free gas (L), and *m*_ts_ is the dry mass of the sample (*g*).

#### 2.2.2. Biochemical Incubation

In this study, 50 g of soil-compost mixture, soil without compost or compost without soil, were added to 200 ml bottles. After adding distilled water, the bottles were covered with caps to avoid gas tightness and placed into the climate chamber at 27°C.

Four parameters were analyzed: O_2_ consumption, DH-ase, HA content, and DOC content on days 0, 5, 10, 20, 40, and 50 of incubation. Reincubation was started after water addition to reactivate biochemical activity, and samples were collected on days 51, 55, 60, 70, and 90. After measurement, the parameters were combined into two parts (0–50 days and 51–90 days) by including the collective days of two stages.

### 2.3. Analyses

Dehydrogenase activity was determined by extraction of iodonitrotetrazolium chloride according to the method described by Von Mersi and Schinner [26]. Humic acids were extracted with a slightly modified procedure with HA, as recommended and used by the International Society for Humic Substances. The sample was extracted with distilled water. Then, 20 mL of distilled water was added to the 2 g sample by a pipette dispenser, giving a ratio between sample and water of 1 : 10. Then, the sample was shaken for 24 hours. The supernatant was removed by centrifugation for 30 minutes at 2000 rpm, followed by filtration through a 0.45 *µ*m membrane. A stock solution of HA (200 mg·L^−1^) was prepared by weighing 0.1 g of HA stock in a 500 mL glass volumetric flask and bringing it to volume with a buffer solution. A series of HA standards were prepared from 0 to 100 mg·L^−1^ through dilution of the stock solution with the buffer solution. The HA content of the samples was measured on a spectrophotometer at a wavelength of 280 nm. The DOC content was determined by water extraction according to the methods described by Jones and Willett [27]. A calibration curve for DOC was prepared from standard solutions that ranged in concentration from 0 to 10 mg·L^−1^, all being diluted from a 100 mg L^−1^ stock solution. The samples were diluted between the calibration series with distilled water, and their DOC content was measured with a DOC analyzer.

### 2.4. Data Analysis

The data were entered into a Microsoft Excel spreadsheet. SPSS version 14.0 was used for statistical analysis, namely, ANOVA one factor tests. Graphs were drawn in SigmaPlot 10, and data were analyzed using linear regression.

## 3. Results

### 3.1. Effect of Organic Substrates and Incubation Time on Respiration

**Figure 1:**
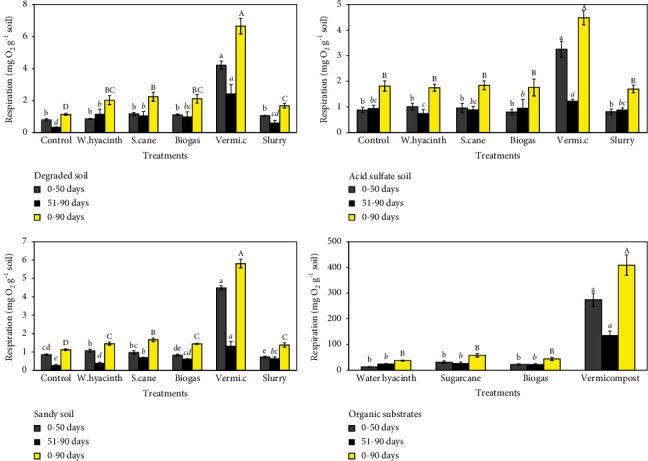
Cumulative respiration in degraded soil, acid sulfate soil, sandy soil amended with water hyacinth (w.hyacinth), sugarcane (s.cane), and vermicompost (vermi.c). The letters indicate significant differences (*p* < 0.05) in respiration within incubation patterns (bars indicate standard deviation).

In the degraded soil, among the treatments, the cumulative O_2_ consumption of respiration varied between 1.14 and 6.64 mg O_2_ g^−1^ soil during 90 days of incubation (Figure 1 top-left). From 0 to 50 days, respiration was the highest in the vermicompost treatment with 4.21 mg O_2_ g^−1^ soil (*p* < 0.05). The other treatments had lower respiration values, ranging from 0.8 to 1.17 mg O_2_ g^−1^ soil, and they did not differ significantly from those of the unamended control. After the samples were dried and subsequently rewetted, microbial respiration was lower than that during the first 50 days. The vermicompost treatment resulted in the highest O_2_ consumption with 2.43 mg O_2_ g^−1^ soil (*p* < 0.05). The treatments amended with biogas, water hyacinth, and sugarcane increased considerably, ranging from 1 to 1.17 mg O_2_ g^−1^ soil compared to the unamended control (0.34 mg O_2_ g^−1^ soil). From 0 to 90 days, all the treatments with amended soil had significantly higher O_2_ consumption than the control. The consumption of O_2_ in the vermicompost treatment (6.64 mg O_2_ g^−1^ soil) was considerably greater than that in the other treatments (*p* < 0.05). The mean respiration rate in soil applied with biogas, water hyacinth, or sugarcane was between 1.68 and 2.24 mg O_2_ g^−1^ soil (*p* < 0.05). The slurry treatment significantly improved O_2_ production with 1.05 mg O_2_ g^−1^ soil (*p* < 0.05) compared to the control treatment.

In the acid sulfate soil, during the incubation period, the cumulative consumption of O_2_ ranged from 1.61 to 4.48 mg O_2_ g^−1^ soil (Figure 1-top-right). After 50 days of incubation period, the cumulative microbial respiration increased substantially in response to vermicompost treatment to 3.25 mg O_2_ g^−1^ soil (*p* < 0.05). No significant differences were observed in the cumulative respiration between the unamended soil and other amendments. After rewetting (51–90 days), the O_2_ respiration rate ranged between 0.74 and 1.23 mg O_2_ g^−1^ soil. There was a significant increase (*p* < 0.05) in O_2_ consumption in the vermicompost-amended soil compared to that in other treatments. The difference in microbial respiration between other treatments and the unamended control was not significant. As an exception, the respiration of water hyacinth added to the soil (0.74 mg O_2_ g^−1^ soil) was significantly lower than that of the soil amended with biogas (0.96 mg O_2_ g^−1^ soil). In general, the vermicompost-amended soil had the highest microbial respiration (4.48 mg O_2_ g^−1^ soil) (*p* < 0.05). When comparing the other amendments (supplements) to the control, no significant change in soil microbial respiration was observed. The consumption of O_2_ ranged between 1.61 and 1.84 mg O_2_ g^−1^ soil. The respiration rate of the second incubation phase showed a strong decrease in the rate of O_2_ consumption in vermicompost-amended soil. There were no differences in microbial respiration among soils amended with water hyacinth, sugarcane, or biogas.

In sandy soil, microbial respiration ranged from 1.13 to 5.81 mg O_2_ g^−1^ soil during 90 days of incubation (Figure 1 bottom-left). There was a higher consumption of O_2_ in the vermicompost treatment (4.49 mg O_2_ g^−1^ soil) than in other amendments (*p* < 0.05). Respiration in the water hyacinth-amended soil was significantly higher than that in the control treatment (*p* < 0.05). The sugarcane and biogas amendments did not show apparent differences in respiration compared to the control. Following the rewetting period of the air-dried soil, the respiration levels of the five amendment treatments differed from the unamended control. Microbial respiration was the highest in vermicompost-amended soil (1.32 mg O_2_ g^−1^ soil, *p* < 0.05). The application of sugarcane to the soil increased respiration, distinguishing it from both biogas and water hyacinth, and yet it remained similar to that of the slurry. Generally, the respiratory activity in amended soils was considerably different from that of an unamended control. Microbial respiration was stimulated in vermicompost soil (5.81 mg O_2_ g^−1^soil), followed by sugarcane application (1.67 mg O_2_ g^−1^soil, *p* < 0.05). The application of water hyacinth, biogas, and slurry produced similar microbial respiration rates with values ranging from 1.38 to 1.46 mg O_2_ g^−1^ soil (*p* < 0.05). When comparing the two phases of incubation, days 0–50 and 51–90, the microbial decomposable activity of the first time phase had a much higher rate than that of the second phase.

In organic substrates, during 90 days of incubation, the respiration activity of all organic substrates ranged between 37 and 408 mg O_2_ g^−1^ substrate (Figure 1 bottom-right). The amount of O_2_ consumption obtained from the treatment applied with vermicompost, sugarcane, biogas, and water hyacinth varied from 274 to 408 mg O_2_ g^−1^, 37.2–57.8 mg O_2_ g^−1^, 21.6–26.2 mg O_2_ g^−1^, and 13.2–31.5 mg O_2_ g^−1^, respectively. The consumption of O_2_ from the vermicompost substrate was the highest than that of other substrates (*p* < 0.05).

### 3.2. Effect of Organic Substrates and Incubation Time on DOC

The highest concentrations of DOC were found in soils treated with sugarcane, biogas, and vermicompost. After rewetting on day 51, most treatments began to show slight increases in the level of DOC, indicating renewed activity of soil microorganisms. Again, during second incubation period, the DOC decreased during incubation.

**Figure 2:**
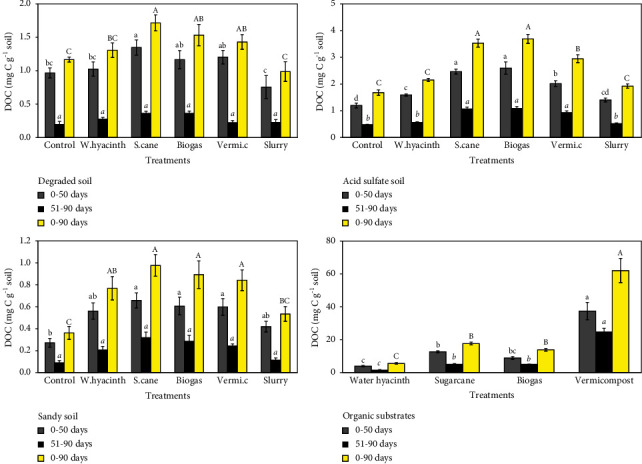
Cumulative DOC in degraded soil, acid sulfate soil, sandy soil amended with water hyacinth (w.hyacinth), sugarcane (s.cane), and vermicompost (vermi.c). The letters indicate significant differences (*p* < 0.05) in respiration within the incubation patterns (bars indicate the standard deviation).

In the degraded soil, the sums of the DOC concentrations ranged from 0.99 to 1.72 mg·C·g^−1^ soil during the entire 90 days of incubation (Figure 2 top-left). Sugarcane treatment significantly increased the quantity of DOC (1.35 mg·C·g^−1^ soil) compared to the control (0.97 mg·C·g^−1^ soil) in the first 50 days (*p* < 0.05). The other treatments did not differ significantly from the DOC in the control. However, the cumulative DOC in the slurry treatment was lower than that in the biogas and vermicompost treatments (*p* < 0.05). After reincubation, there were no significant differences between all treatments with small variations of 0.2 to 0.37 mg·C·g^−1^ soil. The accumulated DOC was lower in the second phase of the incubation than in the first phase (approximately 3 times lower). For 0–90 days, in vermicompost, biogas, and sugarcane treatments, the sum of DOC significantly increased from 1.43 to 1.72 mg·C·g^−1^ soil, whereas treatments amended with slurry and water hyacinth did not affect the content of DOC compared to the control (1.17 mg·C·g^−1^ soil). There were no significant differences in the amount of DOC among the water hyacinth, biogas, and vermicompost treatments.

In acid sulfate soil, the cumulative amount of DOC varied from 1.68 to 3.69 mg·C·g^−1^ soil during the 90-day incubation period (Figure 2 top-right). For 0–50 days, all treated soils showed a significant increase in DOC concentration of 1.59 to 2.6 mg·C·g^−1^ soil compared with that in the unamended control (*p* < 0.05), except for the slurry-amended soil, which reached 1.4 mg·C·g^−1^ soil DOC accumulation. When comparing organically modified soils, the cumulative amount of DOC reached a substantial maximum (*p* < 0.05) in both sugarcane and biogas treatments with values of 2.47 and 2.6 mg·C·g^−1^ soil, respectively. Furthermore, the vermicompost treatment had a significantly higher DOC concentration than water hyacinth and slurry treatments (*p* < 0.05). After rewetting of air-dried soils (51–90 days), cumulative DOC values in vermicompost, sugarcane, and biogas treatments resulted in significantly higher than those of the water hyacinth, slurry, and control treatments (*p* < 0.05). Generally, compared to the control, the addition of sugarcane, biogas, and vermicompost caused a substantial increase in the sum of the DOC concentration from 0 to 90 days (*p* < 0.05). While vermicompost-amended soil had a less significant effect on DOC production compared to both sugarcane- and biogas-amended soils (*p* < 0.05), there were no significant differences in the level of DOC for water hyacinth and slurry-amended soil. Furthermore, at a rate similar to that of degraded soil, the mineralization rate of DOC increased from acid sulfate soil during the second part of the experiment than during the first 50 days.

In the sandy soil, the DOC value changed between 0.36 and 0.98 mg·C·g^−1^ soil during 90 days of the incubation experiment (Figure 2 bottom-left). The results showed that there were no significant differences in the DOC concentration in all soil modified with the treatments from 0 to 50 days after incubation. The soil treated with sugarcane, biogas, and water hyacinth had considerably higher DOC concentration than the unamended control soil (*p* < 0.05). Similarly, there was no significant change among the treatments in the DOC concentration after rewetting the dry soil. Unlike what occurred in primary incubation, there was a decrease in the percentage of DOC during secondary incubation. Compared to those in the amended treatments and unmodified control, the DOC concentration increased significantly in all the organic substrate-treated soils with values ranging from 0.77 to 0.98 mg·C·g^−1^ soil (*p* < 0.05), and the only exception was observed in the slurry-treated soil (0.54 mg·C·g^−1^ soil).

In organic substrates, there was a significant increase in DOC production on the vermicompost substrate to 62 mg·C·g^−1^ substrate at 0–50 days after incubation (Figure 2 bottom-right). However, there was no difference in DOC concentration between the biogas substrate (8.89 mg·C·g^−1^ substrate) and the sugarcane and water hyacinth substrates. On the other hand, the cumulative DOC on the sugarcane substrate (37.38 mg·C·g^−1^ substrate) was significantly higher than that in the water hyacinth (3.99 mg·C·g^−1^ substrate). For 51–90 days and 0–90 days, the vermicompost substrate exhibited the highest DOC concentration, with values of 24 mg·C·g^−1^ substrate and 62 mg·C·g^−1^ substrate, respectively (*p* < 0.05). The DOC concentration of the water hyacinth substrate was lower than that of other substrates at 1.56 mg·C·g^−1^ for 51–90 days and 5.55 mg·C·g^−1^ substrate for 0–90 days. There were no differences in DOC concentration for biogas and sugarcane substrates, which exhibited similar values of 5.01 mg·C·g^−1^ substrate for 51–90 days and respective values of 13.9 and 17.6 mg·C·g^−1^ substrate for 0–90 days.

### 3.3. Effect of Organic Substrates and Incubation Time on Humic Acid (HA)

According to DOC production, humic acid (HA) production was the highest at the beginning of incubation (day 0) and decreased dramatically over the next 20 days. Soon after the rewetting on day 51, the HA content increased in all soils before again decreasing as HA production decreased during the following 50 days.

**Figure 3:**
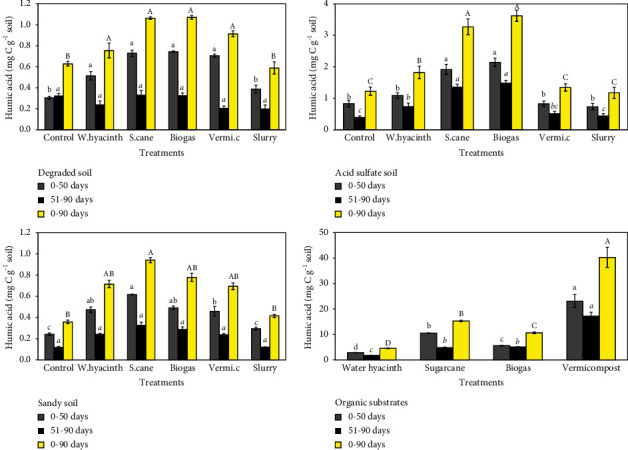
Cumulative humic acid (HA) for sandy soil amended with water hyacinth (w.hyacinth), sugarcane (s.cane), and vermicompost (vermi.c). The letters indicate significant differences (*p* < 0.05) in HA within the incubation patterns (bars indicate standard deviation).

In the degraded soil, during the three months of incubation, the HA concentration ranged from 0.76 to 1.1 mg·C·g^−1^ soil (Figure 3 top-left). At 0–50 days and 0–90 days, the amount of HA was 0.51 or 0.76 mg·C·g^−1^ soil for the water hyacinth treatment, 0.73 or 1.06 mg·C·g^−1^ soil for sugarcane treatment, 0.74 or 1.07 mg·C·g^−1^ soil for biogas treatment, and 0.71 or 0.91 mg·C·g^−1^ soil for vermicompost treatment, respectively. The results indicated that there were significant increases in HA concentration between the other soil modified with other treatments and the control, except for the slurry treatment. At 51–90 days, there were no significant differences observed in HA concentration after rewetting as the HA concentration ranged from 0.2 to 0.33 mg·C·g^−1^ soil among all the treatments.

In the acid sulfate soil, the sum of the HA concentrations ranged from 1.18 to 3.62 mg·C·g^−1^ soil during the whole experiment (Figure 3 top-right). In the first 50 days, the HA concentrations in sugarcane- and biogas-modified soils significantly increased to 1.91 and 2.14 mg·C·g^−1^ soil, respectively. No significant changes in HA concentration were found for other treatments as they ranged from 0.73 to 1.09 mg·C·g^−1^ soil. The concentration of HA decreased in the following rewetting period from 51 to 90 days. In water hyacinth, sugarcane, and biogas treatments, the sum of HA was significantly higher than in the control with 0.73, 1.48, and 1.35 mg·C·g^−1^ soil for 0–50 days and 1.82, 3.27, and 3.62 mg·C·g^−1^ soil for 0–90 days, respectively.

In the sandy soil, the HA concentration varied between 0.54 and 0.94 mg·C·g^−1^ soil during 3 months of laboratory incubation (Figure 3 bottom-left). The sugarcane treatment had the highest HA concentration with 0.62 mg·C·g^−1^ soil in the first 50 days (*p* < 0.05). There were significantly higher amounts of HA concentration in the water hyacinth, biogas, and vermicompost treatments than in the slurry treatment and the control. However, no significant differences in the HA concentration were observed among the treatments after rewetting for 51–90 days. The amount of HA from the sugarcane treatment increased significantly (0.94 mg·C·g^−1^ soil; *p* < 0.05) compared to that from both the slurry treatment and the control at 0–9 days. The other treatments did not differ in their sum of HA.

For the organic substrates, the HA value changed between 4.58 and 40.3 mg·C·g^−1^ during 90 days of incubation (Figure 3 bottom-right). The cumulative HA concentration was the highest in vermicompost (23.1 and 40.3 mg·C·g^−1^ substrate for 0–50 days and for 0–90 days, respectively), followed by sugarcane (10.5 and 15.3 mg·C·g^−1^ substrate for 0–50 days and 0–90 days, respectively). There was a significant decrease in the HA concentration between water hyacinth (*p* < 0.05) at 2.84 (0–50 days) and 4.58 mg·C·g^−1^ substrate (0–90 days), and biogas at 5.57 (0–50 days) and 10.6 mg·C·g^−1^ substrate (0–90 days).

Following the subsequent rewetting stage on day 51, the rate of HA release decreased, as the maximum rate of HA production occurred mainly during the previous stage. The highest concentration of HA was found in vermicompost (*p* < 0.05) at 17.2 mg·C·g^−1^ substrate. Sugarcane (4.82 mg·C·g^−1^ substrate) and biogas (5.06 mg·C·g^−1^ substrate) had significantly increased the HA concentration compared to water hyacinth (1.73 mg·C·g^−1^ substrate).

### 3.4. Effect of Organic Substrates and Incubation Time on Dehydrogenase (DH-Ase) Activity

**Figure 4:**
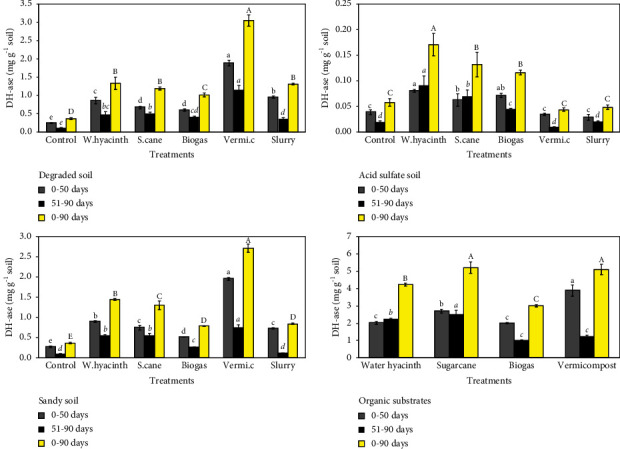
Cumulative dehydrogenase (DH-ase) activity for sandy soil amended with organic substrates: Water hyacinth (w.hyacinth), sugarcane (s.cane), and vermicompost (vermi.c). The letters indicate significant differences (*p* < 0.05) in DH-ase within the incubation periods (bars indicate standard deviation).

In the degraded soil, the sum of the DH-ase activity ranged from 0.37 to 3.05 mg·g^−1^ soil during 90 days of incubation (Figure 4-top-left). In the first 50 days, there were significant differences in DH-ase activity in all treatments except for the sugarcane and biogas treatments. Most treatments with amended soil had significantly higher DH-ase activity than the unamended control (*p* < 0.05). Compared with that in the amended treatments, the DH-ase activity showed a significant increase in all treatments in a sugarcane and biogas < water hyacinth < slurry < vermicompost. After exposure to a drying and rewetting period, most of the soils modified by treatments significantly increased DH-ase activity compared to that of the control. Vermicompost treatment showed the highest DH-ase activity with a value of 1.16 mg·g^−1^ soil (*p* < 0.05). The DH-ase activity of the sugarcane treatment (0.52 mg·g^−1^ soil) was significantly higher than those of biogas and slurry treatments (0.41 and 0.36 mg·g^−1^ soil, respectively). Water hyacinth treatment significantly increased the DH-ase activity (0.48 mg·g^−1^soil) in comparison to slurry treatment only. At 0–90 days, among organic soil amendments, vermicompost-treated soil exhibited the highest DH-ase activity (3.05 mg·g^−1^ soil), followed by soil treated with sugarcane (1.19 mg·g^−1^ soil), slurry (1.32 mg·g^−1^ soil), and water hyacinth (1.34 mg·g^−1^ soil). The biogas-treated soil had the lowest DH-ase activity (1.01 mg·g^−1^ soil) but this value was significantly higher than that of the control (*p* < 0.05).

In the acid sulfate soil, the DH-ase activity varied between 0.04 and 0.17 mg·g^−1^ soil during 90 days of incubation (Figure 4 top-right). The result indicated that the DH-ase activity did not differ between the soil treated with vermicompost or slurry and the control in the first 50 days. The sugarcane treatment (0.06 mg·g^−1^ soil) resulted in significantly lower DH-ase activity than water hyacinth treatment (0.08 mg·g^−1^ soil). However, biogas treatment (0.07 mg·g^−1^ soil) was not significantly different in DH-ase activity compared to sugarcane and water hyacinth treatments. After rewetting, the highest DH-ase activity was found in the water hyacinth treatment (0.09 mg·g^−1^ soil). The DH-ase activity in the sugarcane treatment (0.07 mg·g^−1^ soil) was significantly higher than that in the biogas treatment (0.04 mg·g^−1^ soil). The treatments amended with vermicompost and slurry were close to 0.01 mg·g^−1^soil and showed no significant differences compared to the control (0.02 mg·g^−1^ soil).

In the sandy soil, the DH-ase activity ranged from 0.36 to 2.71 mg·g^−1^ soil during the 90 days (Figure 4-bottom-left). At 0–50 days, all amended treatments significantly increased in DH-ase activity compared with that of the unamended control. The DH-ase activity in the soil treated with slurry and sugarcane (0.73 and 0.75 mg·g^−1^ soil, respectively) was significantly higher than that in the soil treated with biogas (0.52 mg·g^−1^ soil). The DH-ase activity of the water hyacinth and sugarcane-amended soils (0.55 mg·g^−1^ soil) was significantly higher than that in the biogas-amended soil (0.27 mg·g^−1^ soil). However, slurry-amended soil did not significantly increase the activity of the DH-ase enzyme. Most treatments with amended soil had significantly greater DH-ase activity in comparison to the control at 0–90 days. The highest activity of DH-ase was observed in soil (2.71 mg·g^−1^ soil), while slurry-amended soil (0.82 mg·g^−1^ soil) and biogas-amended soil (0.79 mg·g^−1^ soil) had the least value of DH-ase (*p* < 0.05). Water hyacinth-amended soil (1.45 mg·g^−1^ soil) considerably stimulated greater DH-ase activity greater than the sugarcane soil (1.3 mg·g^−1^ soil).

In the organic substrate, the DH-ase activity varied between 3.0 and 5.2 mg·g^−1^ substrate during 90 days of incubation (Figure 4 bottom-right). Vermicompost had the highest DH-ase activity (3.88 mg·g^−1^ substrate in the first 50 days). On the other hand, sugarcane (2.7 mg·g^−1^ substrate) has significantly increased the DH-ase activity compared to water hyacinth and biogas (2.00 and 2.02 mg·g^−1^ substrate, respectively). At 51–90 days, the highest level of DH-ase activity reached 2.51 mg·g^−1^ substrate in sugarcane (*p* < 0.05). Vermicompost with a value of 1.01 mg·g^−1^ substrate and biogas with 1.01 mg·g^−1^ substrate showed significantly lower DH-ase than in the water hyacinth (2.22 mg·g^−1^ substrate). In general, the highest DH-ase activity occurred in vermicompost and sugarcane, which had values of 5.13 and 5.21 mg·g^−1^ substrate, respectively (*p* < 0.05). The DH-ase activity of biogas (3.01 mg·g^−1^ substrate) was significantly higher than that of water hyacinth (4.24 mg·g^−1^ substrate).

## 4. Discussion

### 4.1. Effect of Organic Substrates and Incubation Time on Respiration

The increase in O_2_ consumption after three soils were amended with organic substrates in the experiment could be explained by the improved microbial decomposition of the soil organic matter [28, 29]. Likewise, the application of organic substrates to soils accelerated microbial activity and therefore increased substrate decomposition with the release of CO_2_ [30–32].

In general, the rate of consumption of O_2_ from organic substrate-treated soils, which occurred rapidly during the initial stages of incubation on day 10 and after rewetting on day 70, followed by a relatively linear utilization of O_2_ until the end of both periods. The increase in the respiration rate of O_2_ on day 10 may have been primarily related to the supply of available C and easily decomposable polysaccharides [33, 34].

In fact, the CO_2_ evolution rate is also an indicator of organic matter turnover and soil microbial activity, which are related to the effect of organic substrate amendments [35–37]. The rapid increase in respiration observed in the three soils treated with vermicompost demonstrated higher organic C decomposition than in those treated with the other amendments, most likely due to the high C content (86%) and high C : N ratio of 30 : 1 that may favor microbial activity. The vermicompost substrate alone had the highest O_2_ consumption. As a result, the readily decomposable organic C fraction in the vermicompost residue was higher than that in the water hyacinth, sugarcane, and biogas residues.

Furthermore, for degraded and sandy soils, high amounts of O_2_ consumption were observed during the incubation stages for treatments with water hyacinth, sugarcane, biogas, and slurry. This indicated a readily decomposable organic C fraction in these substrates. Furthermore, microbial respiration varied between modified organic soils and other soils included in this study. For example, when sugarcane was incorporated into sandy soil, the amount of O_2_ consumed was (1.7 mg O_2_ g^−1^ soil) for degraded soil incorporated with sugarcane. Vermicompost applied to the degraded soil led to the highest rate of O_2_ consumption than the other treatments and the other soils. Although O_2_ consumption in the degraded soil amended with slurry (1.1 mg O_2_ g^−1^ soil) showed low O_2_ consumption compared to the acid sulfate soil (1.6 mg O_2_ g^−1^ soil).

The amount of CO_2_ released from organic substrates in soil varies according to the material used [2, 3], as well as the interactions between the organic amendment and the soil [38]. In this study, a calculation of CO_2_-C oxidization for 90 days relative to organic C showed that the stimulation of microbial activity through the application of organic material was stronger for degraded soil (61–80%) than for the other two soils (acid sulfate soil and sandy soil, 44 to 66%). Therefore, the differences in soil properties affected the amount of CO_2_-C emissions.

Air drying and subsequent rewetting during the second stage were expected to stimulate microbial activity to physically breakdown the protected organic fraction. Nevertheless, the decline in microbial respiration in the second stage of the incubation observed in this study was probably caused by depletion of polysaccharides during the incubation, which is consistent with the conclusions of Špaldoňová and Frouz [39].

Except for the vermicompost amendment, the other organic substrate treatments had little or no effect on microbial respiration activity in acid sulfate soil, suggesting that low soil pH and high Fe, Al, and other heavy metals' availability in the acid sulfate soil may be dominant constraints. Decreased soil pH may have reduced soil microbial respiration, as was found by Xiao et al. [32], or the decline in CO_2_ evolution was probably caused by the accumulation of toxic metabolites during incubation [40, 41]. However, this study clearly showed that the addition of organic substrates to acid sulfate soil increased the soil pH (0.3–1.2 units). Some researchers have supported the idea that the application of organic material can increase soil respiration in acidic soils, by increasing the soil pH and soil fertility through improving the nutrient supply for crop production [42–44]. However, respiration in acid sulfate soil was not common in some organic amendments, so soil acidity problems could be corrected by applying limestone or gypsum in the experiment.

### 4.2. Effect of Organic Substrates and Incubation Time on DOC

Dissolved organic carbon is considered to be composed of a wide range of organic compounds, ranging from short-chain acids to large molecules such as fulvic and humic acids. The results demonstrated that increasing the content of organic C can enhance the release of DOC or reduce sorption on the soil. The application of organic substrates to soil had an effect on DOC accumulation.

Sugarcane, biogas, and vermicompost amendments improved the accumulative DOC, exceptions occurred for soils amendment with slurry, and water hyacinth. Therefore, this can be explained by the higher carbon content in soils treated with sugarcane, biogas, and vermicompost, which ranged from 43.8 to 86.2% compared to that slurry (0.3%) and water hyacinth (25.6%), which controlled the effect of the DOC concentration. In most soils, the DOC concentration increased considerably after being supplied with sugarcane, biogas, and vermicompost substrates. Similar results were obtained for increasing the OC content without decreasing the DOC, and there was a positive relationship between the organic C content and DOC release [45–47].

Furthermore, the DOC concentration was the highest at the start of the experiment (day 0) and gradually decreased with time in the later stages. This decrease is mainly attributed to the mineralization of DOC by microorganisms [48]. Furthermore, decreasing DOC resulted in a decrease in release or an increase in the retention of DOC. The reduction in DOC could be explained by the sorption of the soil material during the experimental period [49–51].

In addition, retention is generally thought to be mostly caused by physicochemical processes such as sorption and/or precipitation. The clay texture of the soil property is ideal for maximizing organic C sorption into the solid phase [52]. In addition, both the clay content and Fe oxides control organic C sorption [53, 54]. The decrease in the DOC concentration is perhaps strongly influenced by adsorption and precipitation of Fe and Al oxides [54] and reduced solubility of DOC at lower pH [55]. In contrast, the concentration of DOC increases with increasing acidity or the addition of sulfate as a result of the cleavage of metal ion-organic matter bonds [56, 57]. Therefore, the result could be due to this dual effect of mineral soil properties, on the one hand, favoring primary production in DOC and, on the other hand, because of the increase in soil C content rates with lower pH. In summary, the accumulated DOC concentration increased in the order of sandy soil < degraded soil < acid sulfate soil.

Moreover, DOC production from organic substrates appears to follow the same regulation as DOC production from soil organic matter [58]. However, this study showed that the DOC content of acid sulfate treated with vermicompost was lower than that of acid sulfate treated with sugarcane or biogas. The DOC content in the organic substrates alone was not similar, indicating that the soil properties could affect the changes in DOC as mentioned above. Therefore, organic supplements could alter many biogeochemical processes and affect the retention of DOC due to changes in soil chemical properties, as well as increasing the concentration of DOC [58].

### 4.3. Effect of Organic Substrates and Incubation Time on Humic Acid

Humic acid (HA) from different sources may differ in its biodegradability. They are heterogeneous mixtures of a variety of organic compounds with varying molecular sizes and properties [59]. The incorporation of vermicompost caused slight increases in the accumulation of HA in the degraded soil. The addition of sugarcane increased the HA content in the three soils, while water hyacinth and biogas enhanced the HA concentration only in acid sulfate and degraded soils, demonstrating that the application of different organic substrates affected a variety of chemical, physical, and biological reactions. Similarly, the characteristics of HA may vary greatly depending on the type of material [60].

Furthermore, the mineralization rates of HA in sandy soils tended to be lower than those in the other two soils, while acid sulfate had the highest mineralization rate among the three soils. This shows that HA from different soils has very different chemical compositions and structures [60].

It is difficult to release HA because it binds to colloidal soil surfaces [61]. During the 3 months, the HA concentration reached its highest value on day 0. Afterward, the rate of HA concentration underwent a lag phase, and during the final period after rewetting, the constant trend of HA also declined slightly. This could be explained by the fact that the HA fraction in the soil slowly decomposed, and the undecomposed residues were protected by adsorption to the components of the mineral soil. Similarly, HA is a relatively stable product of organic matter decomposition. Therefore, no increases in HA were observed for some amendments such as water hyacinth, biogas, and vermicompost when added to sandy soil.

Generally, the removal of HA, which may have inhibitory effects on the mineralization of other associated soils can also increase the mineralization rates of the other isolated components. HA formed from organic substrates was different from soil HA because HA produced from organic substrates has comparatively lower concentrations of total acidity and carboxyl and phenolic functional groups than soil HA [62]. HA from organic substrates could be more easily biodegradable than soil HA because of its lower molecular size and aromatic condensation content, which caused organic sole substrates to strongly increase the HA concentration compared to organic soil amendments.

### 4.4. Effect of Organic Substrates and Incubation Time on Dehydrogenase Activity

In this study, it was found that the incorporation of organic amendments into soil stimulated DH-ase activity due to the possible presence of intracellular enzymes in the added material, and it may have also stimulated microbial activity in the soil [63, 64]. For degraded soils, DH-ase activity was estimated by evaluating effective organic addition [65]. The highest level of DH-ase activity for vermicompost amendment in sandy and degraded soils suggested the availability of a large quantity of biodegradable substrates (which was in agreement with the higher content of labile C observed in these soils), and therefore improved microbial activity.

In addition, the application of organic substrates to these soils, e.g., water hyacinth, sugarcane, biogas, and slurry amendments, also led to an enhancement of DH-ase activity. Hence, the activity of soil DH-ase depended on the condition and intensity of the biological conversion of the organic compounds. DH-ase is considered to exist in soils as integral parts of intact cells that functions as a biological catalyst for specific reactions depending on the type of amendment or soil C [66, 67]. For both the degraded and sandy soils, the DH-ase activity reached a maximum at 5 days and sharply decreased after 20 days. The high DH-ase activity in the soils could be explained not only by the neutral pH conditions but also by the organic amendments. However, a small increase in DH-ase activity was observed at 51 days, after which the rate of decrease in DH-ase activity continued until the end of the experiment, probably due to the decreasing availability of more easily decomposable substrates.

Soil properties such as EC, heavy metal toxic compound, and pH can have a negative effect (either jointly or individually) on enzyme activity [68]. However, unlike in degraded soil and sandy soil, the stimulation of DH-ase activity was strongly limited during the incubation period in acid sulfate soil (less than ten times as compared to the other two soils), indicating that the greater inhibitory impact on DH-ase enzyme activity was largely due to the effect of the low soil pH. Thus, pH may be the best explanation for the overall variance in DH-ase activity. Similarly, pH is the best indicator of DH-ase activity [69], and very little enzyme activity is observed below pH 6.6 and above pH 9.5 [70]. However, in acid sulfate soil, DH-ase activity was enhanced after the addition of water hyacinth, sugarcane, and biogas during the experiment. This indicated that the positive effect of organic amendments on soil biological quality is due to stimulation of microbial growth and/or the addition of microbial cells or enzymes with the amendments that counteract soil deterioration caused by some toxic compounds [71].

## 5. Conclusions

The application of organic substrates to soils influences the accumulation of DOC and HA and can stimulate respiration and DH-ase activity. However, different effects can result from the incorporation of organic substrates into soils and vice versa. In addition, organic substrates alone tend to induce greater respiration, DOC, HA, and DH-ase activity compared to soil organic amendments. Generally, O_2_ consumption, DH-ase enzyme, DOC, and HA parameters of both degraded soil and sandy soil exhibited similar patterns, differed strongly from those of the acid sulfate soil. In particular, acid sulfate soil is capable of producing larger amounts of DOC and HA than both degraded and sandy soils, but the DH-ase enzyme occurs in lower quantities in acid sulfate soils than in the other two soils. The application of sugarcane, biogas, and vermicompost in the three soils increased the DOC concentration. Furthermore, sugarcane increases HA production in all three soils, whereas the addition of water hyacinth and biogas stimulates cumulative HA in degraded and acid sulfate soils, and the application of vermicompost enhances HA only in degraded soils. Almost all organic substrates (water hyacinth, sugarcane, biogas, vermicompost, and slurry) applied to degraded and sandy soils increased O_2_ consumption and DH-ase activity, and vermicompost amendment had the greatest effect. However, for acid sulfate soil, only vermicompost amendment increased O_2_ respiration, in contrast to the combined application of water hyacinth, sugarcane, and biogas which effectively increased DH-ase activity. Unlike the production of DOC and HA, the greater inhibitory impact on microbial respiration and DH-ase enzyme activity is probably due to the pH of the soil in acid sulfate soil.

## Figures and Tables

**Table 1:** Characteristics of the soil and organic substrates.

Soil or organic substrates	pH_H2O_	EC (mS cm^−1^)	Total N (%)	C (%)	C : N
Degraded soil	5.21	0.08	0.11	3.07	27.9
Acid sulfate soil	3.15	0.28	0.11	4.44	40.4
Sandy soil	5.98	0.04	0.04	1.21	30.3
Water hyacinth	7.16	3.40	0.99	25.7	25.8
Sugarcane	7.16	4.74	1.90	43.8	23.1
Biogas	6.64	7.09	2.09	46.3	22.1
Vermicompost	6.73	1.56	2.84	86.2	30.4
Slurry	6.96	3.44	0.05	0.30	6.60

**Table 2:** Summary time and parameters of the experiment.

Exp	Number of treatments	*t*_0_	*t*_1_	*t*_2_	*t*_3_	*t*_4_	*t*_5_	Reincubation	*t*_6_	*t*_7_	*t*_8_	*t*_9_	*t*_10_
		Collected and analyzed							Collected and analyzed				
Res	(3 soils × 5 organic substrates) + 3 soils + 4 organic substrates = 22	Measured during incubation							Measured during incubation				
DH-ase		x	x	x	x	x	x		x	x	x	x	x
HA		x		x	x	x	x		x		x	x	x
DOC		x		x	x	x	x		x		x	x	x

## Data Availability

The data that support the findings of this study are available from the corresponding author upon reasonable request.
